# Anticancer Activity of Propolis and Its Compounds

**DOI:** 10.3390/nu13082594

**Published:** 2021-07-28

**Authors:** Ewa Forma, Magdalena Bryś

**Affiliations:** Department of Cytobiochemistry, Faculty of Biology and Environmental Protection, University of Lodz, Pomorska 141/143, 90-236 Lodz, Poland; magdalena.brys@biol.uni.lodz.pl

**Keywords:** propolis, propolis compounds, cancer, cell proliferation, cytotoxicity, apoptosis, autophagy, angiogenesis, metastasis, cancer therapy

## Abstract

Propolis is a natural material that honey bees (*Apis* *mellifera*) produce from various botanical sources. The therapeutic activity of propolis, including antibacterial, antifungal, and anti-inflammatory effects, have been known since antiquity. Cancer is one of the major burdens of disease worldwide, therefore, numerous studies are being conducted to develop new chemotherapeutic agents and treatments for cancer. Propolis is a rich source of biologically active compounds, which affect numerous signaling pathways regulating crucial cellular processes. The results of the latest research show that propolis can inhibit proliferation, angiogenesis, and metastasis of cancer cells and stimulate apoptosis. Moreover, it may influence the tumor microenvironment and multidrug resistance of cancers. This review briefly summarizes the molecular mechanisms of anticancer activity of propolis and its compounds and highlights the potential benefits of propolis to reduce the side effects of chemotherapy and radiotherapy.

## 1. Introduction

Propolis is a natural and sticky material, also known as bee glue, that honey bees (*Apis mellifera*) produce from saps, resins, and mucilages collected from various parts of the plant, such as leaves, flower buds, and tree barks, then mixing them with beeswax and several bee enzymes [1,2]. The word propolis originates from ancient Greek, in which “pro” stands for “at the entrance to” and “polis” for “community” or “city”, indicating that this natural product is used in hive protection and defense [3,4,5]. Honey bees use this natural material to fix damage in the hive (covering the holes and sealing the cracks in the nest), to refine the internal walls, and to maintain constant humidity and temperature in the hive. Moreover, it is used to defend the colony from pathogen microorganisms, parasites, and predators [1,3,5,6,7]. At elevated temperatures, propolis is soft, pliable, and very sticky, while at low temperatures, it becomes hard and brittle; after cooling, it will remain brittle even at higher temperatures [3]. Propolis is characterized by specific herbaceous aromatic scents with various colors, including brown, yellow, green, and red, depending on the source from which it is obtained and the storage time [1,8].

The therapeutic activity of propolis has been extensively explored in traditional medicine throughout centuries and cultures [6]. The ancient Egyptians used it mainly to embalm their cadavers because it prevented bacterial and fungal overgrowth and decomposition [3]. Propolis has been used by humans in different fields, including mainly folk medicine for the treatment of gastrointestinal diseases (i.e., stomach ulcers and buccal infections), wounds, and burns [3,9]. Hippocrates used propolis to cure wounds and external and internal ulcers. Moreover, in the 17th century, British pharmacopoeias listed propolis as an official drug [5]. During World War II, propolis was used as an antibacterial and anti-inflammatory agent [4]. This natural material was also used for other purposes as a constituent of violin varnish by famous Stradivari, Amati, and others [5]. The use of propolis has therefore been developed over time. It reveals biological properties, including antibacterial, fungicidal, antioxidant, immunomodulatory, and anti-inflammatory, among others [6,7,10,11,12,13,14]. Therefore, propolis is currently incorporated into a wide range of complementary health care products, including creams, gels, skin lotions, shampoos, chewing gums, tinctures, throat sprays, cough syrups, lozenges, soaps, toothpaste, and mouthwash preparations [7,15,16].

In addition to the biological properties mentioned above, propolis and its compounds also have an anticancer activity. It has been shown that both propolis extracts and active compounds can affect the key processes for cancer development, i.e., cell proliferation, evading apoptosis, angiogenesis, invasion, and metastasis. In addition, propolis and its components affect the tumor microenvironment and chemosensitize cancer cells characterized by multidrug resistance. Propolis can also be used by patients undergoing chemotherapy and radiotherapy to reduce the side effects of these therapies. The presented review, based on the latest literature, summarizes the molecular mechanisms of the anticancer activity of propolis and its compounds.

## 2. Composition of Propolis

The chemical composition of propolis is diverse and depends on the geographical and botanical origin, i.e., climate factors, plant resources, place of origin, and time in which it was collected by the bees [5,17]. Honey bees collect plant material for propolis production during the warmest hours of sunny days because of the malleability and softness of the resins that are an essential component of propolis. Therefore, in temperate regions, propolis production takes place from late summer until autumn, whereas in tropical regions, honey bees can collect plant material throughout the entire year [6]. The specificity of the local flora is the main factor that determines the chemical composition of propolis and, subsequently, its biological and pharmacological properties [5]. Based on the origin of the propolis plant components, it has been classified into seven major types: 1. poplar (Europe, China, New Zealand, North America, and Southern South America); 2. birch (Russia); 3. Mediterranean (Sicily, Greece, Crete, and Malta); 4. green (South-eastern Brazil); 5. red (Cuba, North-eastern Brazil, and Southeast Mexico); 6. Clusia (Venezuela and Cuba); and 7. Pacific (Okinawa, Taiwan, Indonesia, and Hawaii) [6,18]. Poplar types propolis originate mainly from the bud exudates of *Populus* spp. and mainly contain flavonoids (flavones and flavanones), phenolic acids (cinnamic acid), and their esters. Birch propolis originates from *Betula verrucosa* Ehrh. and also contains flavones and flavonols but is different from poplar propolis. In the Mediterranean region, honey bees mainly collect the resin of *Cupressus sempervirens*, therefore, Mediterranean propolis is rich in diterpenes. Green propolis contains derivatives of phenylpropanoides and diterpenes, chlorophyll and small amounts of flavonoids collected by bees from young tissues and nonexpanded leaves of *Baccharis dracunculifolia*. Contrary to green propolis, its red type is rich in numerous flavonoids (pinobanksin, quercetin, pinocembrin, daidzein), the source of which are resins of *Dalbergia ecastaphyllum*. The Clusia type of propolis contains benzophenones derivatives and originates from the resin of flowers of *Clusia* sp. Other examples of tropical propolis is Pacific propolis characterized by content of C-prenylflavanones [3,18,19]. The chemical composition and biological activities of propolis extracts depend on the type of solvent used for the extraction. The most commonly used solvent for the extraction of propolis is ethanol (particularly at a concentration of 70–75%) [18,20]. Propolis extracts are also obtained by extraction with solvents such as water, ethyl ether, methanol, hexane, chloroform, glycolic and glyceric solution, and seed oil [18,21]. In fact, in pharmaceutical and health care products, propolis is added in the form of ethanolic and aqueous extracts [21]. The available methods of analyzing the chemical composition of propolis and plant materials included in propolis as well as standardization and quality control methods for industrial applications have been described by Bankova and colleagues [22]. In general, propolis is composed of 50–60% of resins and balms, 30–40% of waxes and fatty acids, 5–10% of essential and aromatic oils, 5–10% of pollen, and about 5% of other substances, such as amino acids, vitamins, macro-, and microelements [5,8,18,23]. According to the literature data, more than 300 compounds have been identified in propolis samples of different geographical origins [15,18,20,23]. The major chemical groups found in propolis are flavonoids, aliphatic and aromatic acids, phenolic esters, fatty acids, alcohols, terpenes, β-steroids, alkaloids that include, but are not limited to chrysin, pinocembrin, apigenin, galangin, kaempferol, quercetin, cinnamic acid, o-coumaric acid, p-coumaric acid, caffeic acid (CA), and caffeic acid phenylethyl ester (CAPE) [3,5,15,24]. Flavonoids are the main substances responsible for the pharmacological properties of propolis, while terpenoids are additionally responsible for the odor of propolis [3]. The biological activities of propolis are the results of the interaction between various compounds. Analysis of the activity of each compound alone allows exploration of the molecular mechanisms underlying the pharmacological properties of propolis [23]. Table 1 summarizes the results of recent in vitro and in vivo studies on the influence of propolis and its active compounds on the processes related to cancer development.

## 3. Mechanisms of Anticancer Activity of Propolis and Its Compounds

### 3.1. Antiproliferative and Cytotoxic Activities of Propolis and Its Compounds on Cancer Cells

A characteristic feature of all neoplastic foci is unlimited cell proliferation. Many, if not all, human cancers fail to regulate and control the cell cycle, resulting in uncontrolled cell proliferation. Proliferation is an important element in the development and progression of neoplastic lesions. This process is associated with disturbances in the expression and activity of proteins in the cell cycle, as well as with altered signaling in many cellular pathways [47,48,49].

The cell cycle is controlled by several mechanisms to ensure proper cell division. Cyclins regulate the cell cycle by binding to and activating cyclin-dependent kinases (Cdks). Phosphorylation of specific targets by cyclin–Cdk complexes trigger processes that activate the cell cycle at the right moment. In eukaryotic organisms, the stages of the cell cycle are divided into two main phases: interphase and mitosis (M phase). During interphase, the cell grows and copies its genetic material. During the M phase, a cell divides its cytoplasm and its DNA into two sets to form two new cells. Preparation for division takes place in the three stages G1, S, and G2. Collectively, the G1, S, and G2 phases are known as the interphase. Cells in the G1 phase may, before engaging in DNA replication, enter a quiescent state called G0. Cells in G0 make up the majority of non-growing, non-proliferating cells [50,51,52,53,54].

Some of the published research papers only state the effect of propolis and its components on the inhibition of the proliferation process without penetrating the molecular mechanism [9,16,55,56,57,58,59,60,61,62,63,64,65]. Nevertheless, there are also research results that bring these mechanisms closer.

It is currently assumed that propolis and its components are reported to modulate cell cycle regulators like cyclin D, cyclin-dependent kinases Cdk-2/4/6, and cyclin-dependent kinase inhibitors, thereby arresting the progression of the cancer cell cycle (G2/M phase), as well as in stage G0/G1, by upregulating p21 and p27 expression [49,63,66]. Ethanol-extracted Cameroonian propolis increased the amount of DU145 and PC3 cells in G0/G1 phase, down-regulated cell cycle proteins (CDK1, pCDK1, and their related cyclins A and B) in both DU145 and PC3 cells, and CDK2 and pCDK2 proteins were down-regulated only in PC3 cells [67].

CA and CAPE induce S-phase cell cycle arrest in a dose- and time-dependent manner in breast cancer cells MDA-MB-231. A dose-dependent decline was also observed for the G0/G1 phase (CAPE), as well as elimination of phase G2/M (CAPE and only mild effect for CA) [35].

Caffeic acid phenylethyl ester has been shown to inhibit the S6 beta-1 ribosomal protein kinase (p70S6K), an intermediary responsible for protein synthesis in the PI3K/AKT pathway and some AKT signaling networks, leading to inhibition of proliferation of prostate cancer cells LNCaP, DU-145, and PC-3 [3]. CAPE and artepillin C (ArtC) dock into and abrogates mortalin-p53 complexes, causing the activation of p53 and the growth arrest of HT1080 (human fibrosarcoma), A549 (human lung carcinoma), and U2OS (human osteosarcoma) cancer cells [32]. Ren and colleagues (2019) showed that CAPE also inhibits the proliferation of HEp2 human epithelial cells by Stat3/Plk1 pathway and inducing S phase arrest [37].

Genistein, another component of propolis, inhibits the cell cycle in the G2/M phases. This is achieved by reducing the expression of cyclin B and inducing p21 in a p53-independent manner, as demonstrated by studies on prostate cancer cells [3].

Claudin-2 is involved in neoplastic proliferation and is highly expressed in human lung adenocarcinoma cells. The expression of this protein is regulated at the stage of transcription and post-translationally. The transcriptional activity of claudin is decreased by the inhibition of mitogen-activated protein kinase kinase (MAPKK)/extracellular signal-regulated kinase (ERK)/c-Fos, and phosphatidylinositol-3 kinase (PI3K)/Akt/nuclear factor-κB (NF-κB) pathways [68,69]. CAPE decreases claudin-2 expression by two different mechanisms. CAPE decreases the level of p-NF-κB and increases that of IκB (inhibitor of NF-κB). The inhibition of NF-κB may be involved in the decrease of claudin-2 mRNA level [70].

A poly-isoprenylated benzophenone named nemorosone (Nem) is the main phytocompound present in Cuban brown propolis (CBP). This compound drives a high percentage of hepatocellular carcinoma HepG2 cells to the G0/G1 phase [46].

The standardized ethanolic extract of poplar-type propolis from Turkey induces cell cycle arrest in MCF7 (breast cancer), HGC27 (human gastric carcinoma), and A549 cancer cell lines by promoting cell cycle arrest in G1/S transition as well as increasing expression rates of cell cycle checkpoint proteins. The 3-O-methylquercetin, chrysin, caffeic acid, CAPE, galangin, and pinocembrin were the main components of this extract. Studies on MCF7, HGC27, and A549 neoplastic cells have shown that propolis has the effect of arresting the cell cycle in the G0/G1 phase by activating p21 [71].

It is known that the intensity of the glycolysis process is associated with a decrease in the activity of critical glycolytic enzymes, which stimulates proliferation. Chinese poplar propolis has been shown to significantly reduce the level of glycolysis at the stage of action of hexokinase 2 (HK2), phosphofructokinase (PFK), muscle isozyme pyruvate kinase M2 (PKM2), and lactate dehydrogenase A (LDHA) in in LPS-induced inflammation [72].

The inflammatory microenvironment plays an important role in carcinogenesis. TLR4 is the most studied member of the toll-like receptor family, which participates in innate immunity. Aberrant expression of TLR4 was also observed in many types of cancer. This receptor can induce chronic inflammation in the tumor microenvironment and lead to stimulation of proliferation and apoptosis suppression of cancer cells [73]. Chinese propolis, as well as CAPE, inhibits breast cancer cell proliferation in the inflammatory microenvironment by inhibiting the Toll-like receptor 4 (TLR4) signal pathway [74]. With respect to esophageal cancer cells, it has been shown that TLR4 activation stimulates cell proliferation via the TLR4-MyD88-TRAF6-NF-κB signaling pathway and that inhibition of NF-κB leads to inhibition of proliferation [75].

**Figure 1:**
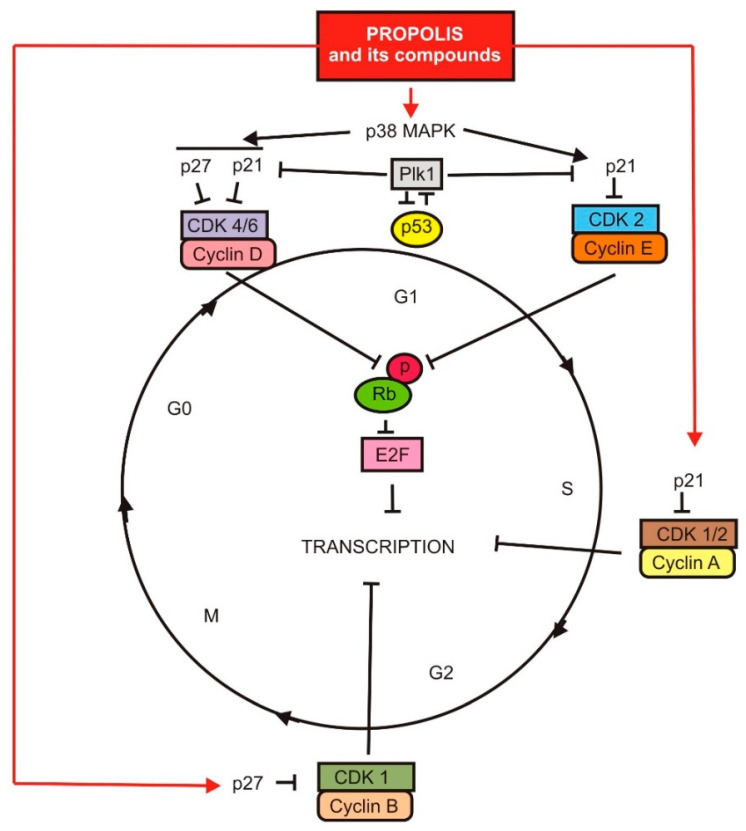
The influence of propolis and its components on cell proliferation.

Vestitol, a Brazilian red propolis bioactive isoflavonoid, down-regulates the alpha-tubulin, tubulin in microtubules, and histone H3 genes. Alpha-tubulin and tubulin in microtubules move descendant chromosomes in cell division during mitosis. The disruption of microtubules in mitosis may, in turn, affect the progression of the cancer cell cycle. The histone H3 is located in the nucleosome and is a unit of the chromatin. This structure maintains the DNA condensation in the cell during mitosis and is essential for the correct separation of sister chromatids and the formation of two daughter cells [31]. Figure 1 shows a schematic visualization of the depicted signaling pathways and their main elements.

The mechanisms of cytotoxic action of antitumor compounds are most often related to apoptosis, cell cycle, and metastasis. The results of studies on the cytotoxicity of propolis and its compounds concerning neoplastic cells are presented below. The MTT test is the most frequently used test to analyze the metabolic activity of a cell and evaluate its cytotoxic activity [76,77].

Brazilian green propolis displays cytotoxic activity against AGP-01 gastric cancer cells. Artepillin C and p-coumaric acid are two of the major compounds that contribute to these activities [33]. Brazilian red propolis extract has a cytotoxic effect against colon cancer cell lines, bladder cancer cells (T24), and prostate cancer cells (PC-3) [23,78,79]. DMEM extracts of Turkish propolis exerts cytotoxic effects on MDA-MB-231 cells [80]. In turn, ethanolic extract of Turkish propolis has been shown to have cytotoxic activity towards hormone-resistant prostate cancer PC-3 cells [81]. Egyptian propolis ethanolic extract prompted cytotoxic effects in HCT-116 (colon cancer), MDA-MB-231, MCF-7, and HeLa (cervical cancer) cancer cell lines [82]. Mexican brown propolis ethanolic extract cytotoxic activity was evaluated in a rat C6 glioma cell and human cervical cancer cell lines (HeLa, SiHa, and CaSki) [56]. The cytotoxic effect on C6 glioma cells is also demonstrated by the ethanolic extract of Turkish propolis [83].

Propolis and its compounds exhibited a time- and dose-dependent cytotoxic effect on human laryngeal epidermoid carcinoma. CAPE-like analogs inhibit the growth of oral submucosa fibroblasts and the growth of tongue squamous cell carcinoma cells. Quercetin, another flavonoid in propolis, has anti-cancer properties. Quercetin inhibits chemically induced oral carcinogenesis in rats [16]. Among the ingredients of propolis, CAPE expressed cytotoxic activity against AGS (human adenocarcinoma of the stomach cells), HCT116, and HT29 (colorectal adenocarcinoma) tumor cells [38].

### 3.2. The Influence of Propolis and Its Compounds on the Apoptotic and Autophagy Process in Cancer Cells

Cell death by apoptosis is an important suppressor mechanism of a neoplastic focus, regardless of the stage of the neoplastic process [84]. However, the latest research results shed new light on this mechanism. It turns out that apoptosis may also have oncogenic potential [85,86]. Therefore, it is very important to understand in detail the signaling pathways induced by propolis and its components leading to apoptosis in neoplastic foci. Only then will it be safe to use propolis in the treatment of neoplastic diseases. When analyzing the literature data, it should be taken into account that the effect of propolis as a whole may be different than that of its components tested separately.

Apoptosis induction by propolis and its compounds coming from different geographical regions has been proven in many studies [19,55,59,61,67,79,82,87,88,89,90,91,92], while in this review, we want to focus strictly on the demonstrated molecular mechanisms of this process.

Apoptosis has been suggested as a program of cellular suicide in which the cell destroys itself to maintain tissue homeostasis. This process is recruited through three different pathways: the extrinsic pathway, the intrinsic pathway, and the granzyme B-dependent pathway. Among these pathways, the intrinsic and extrinsic pathways are the major mechanisms. The intrinsic (mitochondrial pathway) is triggered by different stimuli such as DNA damage, cytokine withdrawal, and endoplasmic reticulum stress. The extrinsic pathway is triggered by a family of death receptors located on the cell membrane [75,93,94,95,96,97].

**Figure 2:**
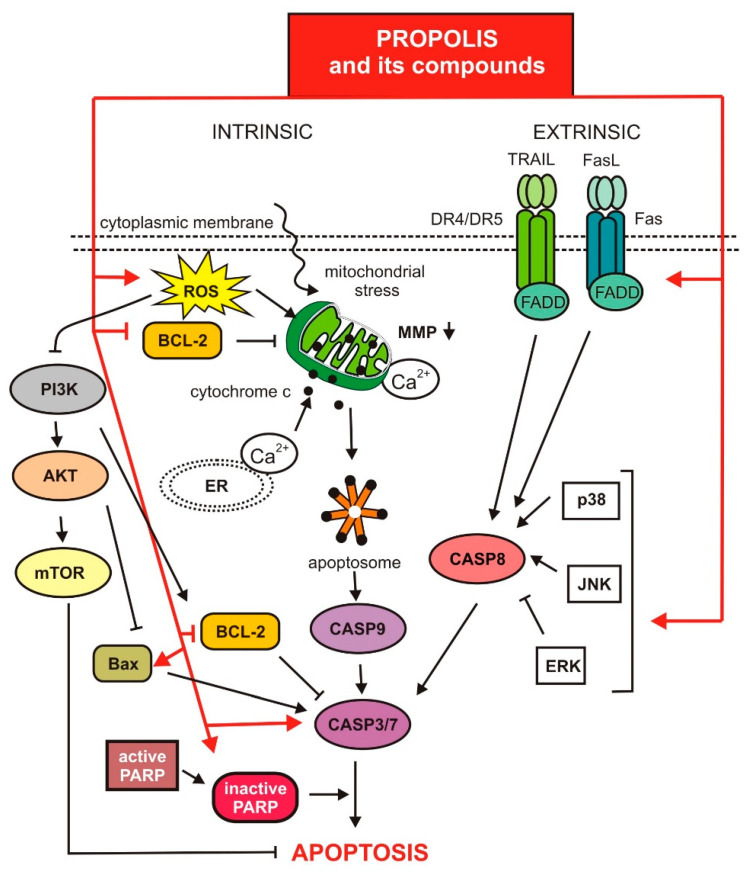
Regulation of apoptosis via propolis and its compounds.

It has been proven that propolis and its components show pro-apoptotic activity by inducing both of the above-mentioned mechanisms. Figure 2 visualizes the molecular signaling pathways involved in the induction and regulation of apoptosis, discussed below.

Artepillin C, a prenylated derivative of p-coumaric acid, is a phenolic compound of Brazilian green propolis [98,99]. This compound induces apoptosis, as revealed by DNA fragmentation, and increases in cleaved caspase-3 and poly ADP-ribose polymerase in castration-resistant prostate cancer (CRPC) CWR22Rv1 cells [34]. Cell death induced in oral squamous cell carcinoma cells by ArtC, at least in part, was a result of a decrease in survivin levels. Survivin is a member of the inhibitor of apoptosis family and is overexpressed in most human tumors, but undetectable in normal adult tissues [100].

Caffeic acid phenethyl ester is an active component of propolis. It has pro-apoptotic activity through the caspase-3/7 pathway. CAPE significantly increased apoptosis mediated by TRAIL (tumor necrosis factor-related apoptosis ligand inducer) by positive regulation of DR5 (death receptor 5) mediated by CHOP (C/EBP family transcription factor) [36,38,49,63]. CAPE also affects the apoptotic intrinsic pathway by increasing ROS production and also decreases expression of apoptosis inhibitors such as transporter associated with antigen processing 1 (cTAP-1), baculoviral IAP repeat-containing protein3 (cIAP-2), and X-linked inhibitor of apoptosis protein (XIAP) [3,63]. Research has also shown that CAPE induces apoptosis by decreasing the levels of proteins related to carcinogenesis, including Akt, glycogen synthase kinase 3 beta (GSK3b), class O forkhead box transcription factor 1 (FOXO1), FOXO3a, NF-kB, S-phase kinase-associated protein 2 (Skp2), and cyclin D1 [16,101,102].

Chrysin is a component of propolis, whose pro-apoptotic activity and its molecular mechanism are well understood. This compound has been shown to initiate apoptosis via the mitochondrial pathway. It was found that chrysin caused a loss of mitochondria membrane potential (MMP) while increasing the production of reactive oxygen species (ROS), cytoplasmic Ca^2+^ levels, and lipid peroxidation [103].

Chrysin induced ROS production, which lowered the phosphorylation of protein kinase B and the mammalian target of rapamycin (mTOR). Chrysin also induced endoplasmic reticulum (ER) stress by activating unfolded protein response proteins (UPR) such as PRKR-like ER kinase (PERK), eukaryotic translation initiation factor 2α (eIF2α), and 78 kDa glucose-regulated protein (GRP78). Research results also indicate the involvement of the PI3K/Akt and MAPK pathways in chrysin-induced apoptosis [25,63,104,105].

Another component of propolis whose molecular mechanism of pro-apoptotic action has been investigated is galangin, which significantly reduced apoptosis and autophagy dose-dependently in mice bearing B16F1 melanoma tumor cells [27]. It was found that in human laryngeal cancer cell lines, galangin reduces the expression of Bcl-2 protein and may perform a suppressor function by inhibiting the PI3K/AKT signaling pathway [28]. Galangin also induces cell apoptosis via the activation of p38 mitogen-activated protein kinase (p38 MAPK) and significantly improves TRAIL (death receptor) mediated apoptosis [63,106].

Quercetin, a flavonoid present in propolis, induces apoptosis and necrosis via reduced expression of thymidylate synthase (TS), a key S-phase enzyme, in a time- and concentration-dependent manner. Quercetin also induces caspase-3-dependent apoptosis [16,107].

Chrysin, caffeic acid, p-coumaric acid, and ferulic acid, polyphenols of propolis, induce PRODH/POX-dependent apoptosis through up-regulation of mitochondrial proline degradation and down-regulation of proline utilization for collagen biosynthesis [16,26,63].

Artepilin C, baccharin, and drupanin, a propolis derivative, had a potent apoptosis-inductive effect even on drug-resistant colon cancer cells. Mostly baccharin, plus drupanin, synergistically inhibited growth by activating the internal and external apoptotic signaling pathway via TRAIL/DR4/5 and/or FasL/Fas and by increasing the expression of miR-143, which decreased the expression of MAPK/Erk5 and its downstream target c-Myc in human colon cancer cell lines [63].

Concerning the ethanolic extract of propolis (EEP), broad pro-apoptotic activity was demonstrated. EEP has been shown to activate the apoptosis process through the tumor necrosis factor-related apoptosis-inducing ligand (TRAIL) pathway, the cellular tumor antigen p53, Bcl-2-associated X protein (Bax) transcriptional activation, and inhibition of the extracellular signal-regulated kinases (ERK) 1/2 signaling pathway [3,39,63].

CAPE activated Bax protein cause it to undergo a conformational change, translocate to the mitochondrial outer membrane and oligomere. CAPE also significantly increased PUMA expression, which colocalizes with Bax in human oral cancer cell lines [39,63].

Special Chinese propolis sourced from the Changbai Mountains (CBMP) in Northeast China causes cell apoptosis in human gastric cancer cells with increased production of reactive oxygen species (ROS) and reduced mitochondrial membrane potential. After treatment, the pro-apoptosis proteins Bax and Bid are upregulated, while the anti-apoptosis protein Bcl-2 is downregulated. The release of cytochrome C from mitochondria to the cytoplasm is observed, as well as the activation of cleaved caspases (8, 9, and 3) and PARP. Meanwhile, pro-apoptotic protein TP53 is also activated after CBMP treatment [65].

Cuban red propolis (CP) and Brazilian green propolis (BP) extracts influence the production of reactive oxygen species and decrease mitochondrial membrane potential. Extracts induce apoptosis by activating TP53, CASP3, BAX, p21 signaling and downregulating BCL2, BCL-XL, and PUMA. But in a similar study, researchers found that Brazilian propolis did not affect BCL-2, BCL-XL, NOXA, and PUMA expression [108,109].

Propolis triggers colon cancer cell death by increasing DNA condensation, which accounts for the induction of apoptosis [63].

Autophagic cell death is considered to be one type of programmed cell death and interacts closely with apoptosis. This process is discussed in detail in new review papers [110,111,112]. LCI, LC3II, and Beclin 1 are required for the autophagy-mediated elimination of unfolded ubiquitinated long half-life proteins. The precursor form of LC3 is modified into LC3-I and LC3-II. LC3-I is localized in the cytosol, and LC3-II is membrane-associated and a key hallmark for autophagosome formation [110].

Artepillin C showed high autophagy-inducing activity accompanying LC3-II upregulation in CRPC CWR22Rv1 cells [34].

After treatment of the laryngeal cancer cells with galangin, the presence of LC3-II was found, indicating the development of the autophagy process. It has been suggested that galangin, through this mechanism, stimulates the autophagy process and cell death in human laryngeal carcinoma [28].

Chinese propolis and its constituent CAPE induce autophagy in breast cancer cells. Ethanol-extracted Chinese propolis and CAPE increased expression of LC3-II and decreased p62 level to induce autophagy [72,74].

This review aims to summarize the results of studies on the mechanism of activity of propolis and its active compounds in the apoptotic and autophagy process. Our review shows that propolis and its presented compounds induce apoptosis pathways in cancer cells and may be useful as potential chemotherapeutic or chemopreventive anti-cancer drugs.

### 3.3. Propolis and Its Compounds of Anti-Angiogenic Activity

In the process of angiogenesis, new blood vessels are created from existing ones. There are three types of angiogenesis—sprouting angiogenesis, intussusception or splitting angiogenesis, and looping angiogenesis. These processes have been thoroughly described and analyzed in many articles [113,114]. It is assumed that the balance between stimulation and inhibition of new vessel formation is disturbed in neoplastic foci due to the predominant influence of pro-angiogenic factors [113,115,116].

**Figure 3:**
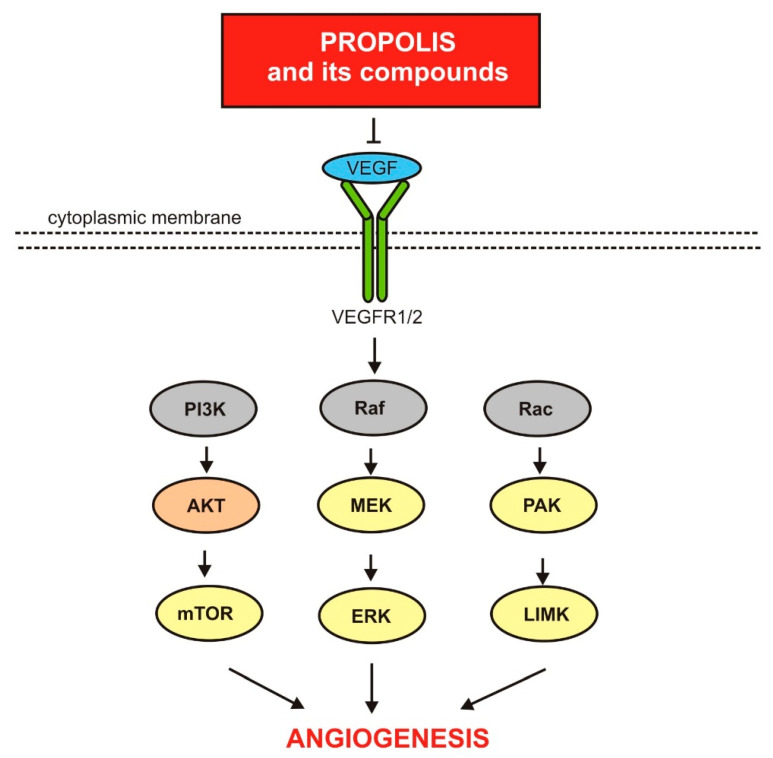
Anti-angiogenic activity of propolis and its compounds.

Experimental data show that propolis and some of its components show anti-angiogenic activity against neoplastic cells [1,4,117]. The molecular basis of this activity is provided below. Figure 3 presents a schematic model of the molecular mechanisms by which propolis and its components influence the angiogenesis process.

Caffeic acid can act on angiogenesis by reducing the phosphorylation of JNK-1 (c-Jun N-terminal kinases) via decreasing the activation of HIF-1α (hypoxia-inducible factor 1). The effect of these mechanisms is the reduction of vascularisation induced by VEGF (vascular endothelial growth factor) and consequently suppressing tumor growth. CA also reduces hepatocarcinoma cell angiogenesis through blocking STAT3 (transcription factor and signal translation 3) signaling and suppress MMP2 and MMP-9 (collagen IV metalloproteases) [36].

Propolis and CAPE directly inhibit VEGF production, as well as inhibiting metalloproteinase 2 and 9 production [16,63].

It has been suggested that the anti-angiogenic activity of propolis concerns the downregulation of the activity of cell signaling pathways mediated by Jun N-terminal kinase, ERK1/2, NF-kB, Akt, and PAK1. It should be added that propolis is a source of PKA1 inhibitors such as nymphaeols A and C, frondoside A, and artepillin C [49,63,118].

### 3.4. Anti-Metastatic Activity of Propolis and Its Compounds

Metastasis is the most complex of processes that leads to the development of secondary tumors located in tissues/organs distant from the primary tumor [119]. The epithelial-mesenchymal transition (EMT) plays a key role in metastasis [120]. This mechanism is discussed in detail in many review articles [120,121,122,123,124]. Cell motility, migration, and invasion are also regulated by Notch, Wnt, and Hedgehog signaling pathways [125].

Numerous studies conducted in recent years have reported that propolis and its active compounds (CA, CAPE, artepillin C, nemorosone) inhibit the migration and invasion of cells of many types of cancer, including glioblastoma multiforme (U87MG), prostate cancer (Du145, PC3), breast cancer (MCF-7, MDA-MB-231), fibrosarcoma (HT1080), osteosarcoma (U2OS), lung cancer (A549), and colorectal cancer (HT-29, LoVo) [19,32,67,74,88,126,127,128,129].

Propolis and its components influence the activity of signaling pathways crucial for metastasis. A study conducted on a mouse model of breast cancer showed that propolis could inhibit tumor progression. Administration of propolis to mice suppresses the expression of Wnt2 and FAK (focal adhesion kinase) proteins in mammary gland cancer induced by DMBA (7,12-dimethylbenanthracene) [130]. Wnt2 and FAK overexpression has been found in many types of cancer. Both proteins play an important role in the promotion of cancer cell growth and migration [131,132].

Treatment of breast cancer cells (MCF-7 and MDA-MB-231) with CAPE negatively regulates the expression of mortalin at both protein and transcript levels. Mortalin is overexpressed in cancer cells and promotes carcinogenesis and metastasis. Moreover, CAPE-treated cells were characterized by decreased expression of vimentin, MMP-2, MMP-9, β-catenin, TGFβ, and Wnt 3α, which are key regulators of cell migration [40]. Tseng and colleagues [41] demonstrated that CAPE suppressed the migration and invasion of PC-3 and DU145 prostate cancer cells and reduced the metastasis of PC-3 xenografts in a mice model. CAPE treatment of prostate cancer cells induced ROR2 (receptor tyrosine kinase-like orphan receptor 2) and Wnt5a expression, which are involved in the non-canonical Wnt signaling pathway. Moreover, CAPE downregulated expression of a protein crucial for EMT, such as NF-κB p65, MMP-9, Snail, β-catenin, and decreased phosphorylation of IκBα [41].

Propolis and its components modulate the ability of cancer cells to migrate and invade through MAPK and PI3K/AKT signaling pathways [28,30,42,43,108]. The c-prenylflavonones, including propolin C, are specific active compounds of propolis from East Pacific regions, such as Taiwan and Okinawa [30]. Propolin C causes a dose-dependent inhibition of ERK and AKT phosphorylation in the HCC827 lung cancer cell line. After propolin C treatment, expression of E-cadherin (epithelial-like cell marker) was upregulated, while expression of vimentin (mesenchymal-like cell marker) and Snail (prominent inducer EMT) were downregulated in a dose-dependent manner [30]. Galangin, one of active compounds of propolis, belongs to the flavonol class of flavonoids. Galangin-treated human laryngeal cancer cells (Tu212 and HEP-2 cell lines) showed decreased migration and invasion ability. Galangin also caused downregulation of Ras, Raf, and PI3K protein expression and inhibition of p38, AKT, and NF-κB phosphorylation [28]. CAPE represses nasopharyngeal carcinoma (TW01 and TW04 cell lines) and oral squamous cancer (SAS and OECM-1 cell lines) cells’ invasion and EMT by stimulatingNDRG1 (N-myc downstream regulated 1) expression. NDRG1 belongs to a family of N-myc downstream-regulated genes (NDRG1-NDRG4). This protein is involved in the suppression of migration and epithelial-mesenchymal transition by regulating N-cadherin, E-cadherin, vimentin, Snail, and Slug protein levels. Interestingly, CAPE treatment of nasopharyngeal and oral cancer cells increased phosphorylation of ERK, JNK, and p38 in a dose- and time-dependent manner. The use of MAPK inhibitors led to partial inhibition of NDRG1 induction by CAPE [42,43]. Moreover, CAPE also repressed the activity of STAT3, whose overexpression and constitutive activation play a role in the oncogenesis of nasopharyngeal cells [43].

The study of Fraser and colleagues [44] showed that CAPE can regulate invasion and migration of breast (MDA-MB-231, MDA-MB-468), colon (SW620) and lung (H460) cancer cells by blocking voltage-gated sodium channel (VGSC) activity. VGSCs are overexpressed in many types of cancers and promote metastasis [44].

The polymorphonuclear neutrophils (PMNs) infiltrate into the desmoplastic stroma of pancreatic ductal adenocarcinoma. This process may create a proinflammatory microenvironmental and stimulate cancer progression [45]. PMNs contain human neutrophil elastase (HNE), which induce cell growth and migration of pancreatic ductal carcinoma cells (PANC-1). CAPE inhibited the migration of PANC-1 cells induced by HNE. In silico analysis showed that CAPE directly bound to the binding pocket of HNE and inhibited the activity of this protease [45].

Tumor-associated macrophages (TAMs) play a pivotal role in immunosuppression and carcinogenesis. Treatment of HepG2 (hepatocellular carcinoma cells) with supernatant of human monocytic THP-1 cells and HT-29 (colorectal adenocarcinoma cells) with supernatant of M2-like macrophages or co-culturing both types of cells stimulated migration and invasion of studied cancer cells. Interestingly, nemorosone and/or Cubian brown propolis extract treatments decreased migration and invasion, both in the presence and absence of conditioned medium or M2-like macrophages [46,64]. Cuban propolis and nemorosene treated M2-like macrophages were characterized by reduced mRNA levels of proinflammatory factors, such as IL-8, IL-10, CCL2 and VEGF. Additionally, the treatment with Nem and Cuban propolis significantly downregulated the activity of MMP-9, which is released by M2-like macrophages. MMP-9 contributes to cancer cell infiltration and invasiveness [64].

**Figure 4:**
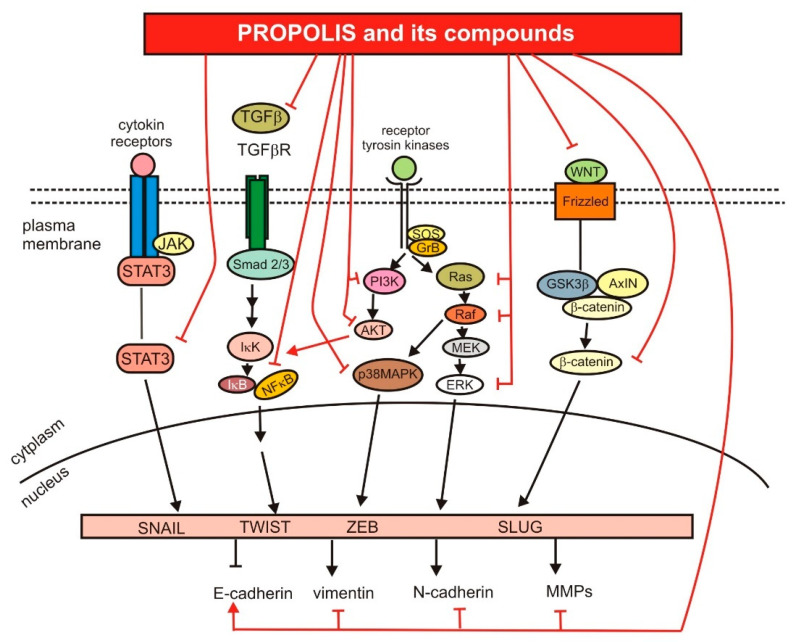
The role of propolis and its compounds in the regulation of signaling pathways crucial for metastasis.

The graphic summary of the impact of propolis and its compounds on the metastasis process is presented in Figure 4.

## 4. The Use of Propolis and Its Components in Cancer Therapy

Numerous studies have evaluated the biological effects of natural products in cancer therapy. Natural substances and their derivatives are used as chemotherapeutic agents, including vincristine, vinblastine, and taxanes (paclitaxel and docetaxel). Moreover, natural compounds may protect healthy cells from the damage caused by chemotherapy and radiotherapy, and limit the more severe effects of anticancer therapy [133].

Motawi and colleagues [134] studied how tamoxifen, CAPE, and their combination effect tumor size, survival time, and life span of Ehrlich tumor-bearing mice. A combination of tamoxifen and CAPE increased the life span of tumor-bearing mice two-fold compared with those treated with tamoxifen or CAPE alone. A combination of studied substances significantly decreased the tumor size and weight compared with the control group and tamoxifen-treated mice [134].

Propolis may also affect the effectiveness of chemotherapy with cytotoxic drugs. Sameni and colleagues showed in a mouse model of colorectal cancer that administration of Iranian propolis extract in combination with 5-fluorouracil (5-FU) significantly reduced the number of azaxymethane-induced aberrant crypt foci compared to 5-FU or propolis alone. Moreover, the propolis combined with 5-FU decreased the expression of Cox-2, iNOS, and β-catenin proteins, which play an important role in the incidences and progression of colorectal cancer [135].

Propolis may also have a positive effect on the efficacy of photodynamic therapy (PDT). PDT is a clinically approved form of therapy involving photosensitizing chemical substances (such as protoporphyrin IX) and a light that activates photosensitizers accumulated in cancer cells. Brazilian green propolis extract significantly enhances the intracellular accumulation of protoporphyrin IX (PpIX) in human epidermoid carcinoma cells A431 and increased PpIX-mediated photocytotoxicity in a xenograft model [136].

Chemotherapy and radiotherapy are the most widely used treatments for human cancer, and both are associated with many side effects [137,138]. Darvishi and colleagues [137] analyzed the antioxidant and anti-inflammatory effects of propolis during a randomized, double-blind clinical trial study on breast cancer patients who revived chemotherapy. In the group of patients taking propolis, in contrast to the placebo group, no increase in the level of proinflammatory cytokines (TNFα, IL-2) and protein carbonyl (biomarker of oxidative stress) was observed [137]. Propolis also shows the radio-protective effects in the case of chemotherapy receiving breast cancer patients undergoing radiotherapy. Moreover, breast cancer patients undergoing radiotherapy and supplemented with propolis had a statistically significant longer median disease-free survival time than the control group (radiotherapy without propolis supplementation) [138].

Oral mucositis is a major side effect of chemotherapy and radiotherapy [139,140]. Piredda and colleagues [140] showed that propolis was safe and well-tolerated by breast cancer patients receiving chemotherapy. Mouth rinsing with dry extract of propolis was effective in the reduction of significant symptoms of oral mucositis in patients with breast cancer during chemotherapy [140]. Similar results were also obtained in patients receiving chemotherapy for head and neck cancer [141]. The meta-analysis conducted by Kuo et al. [139] confirmed that propolis mouthwash is effective and safe in the treatment of chemo- or radiotherapy-induced oral mucositis in cancer patients.

Multidrug resistance (MDR) is also a significant problem in cancer therapy. MDR is the cellular mechanism by which patients’ cancer cells develop resistance to unrelated chemotherapy drugs [142,143]. Doxorubicin (DOX) is one of the drugs commonly used in the treatment of many types of cancer, including breast, lung, ovarian, bladder, gastric, and thyroid cancer [144,145]. Propolis resulted in the inhibition of proliferation of DOX-resistant lung cancer cells (A549) [146]. P-glycoprotein (P-gp) is a multidrug membrane transporter, which effluxes out chemotherapeutic drugs from the cancer cells [142,143]. Kebsa et al. showed that propolis inhibited the P-gp efflux pump in a dose-dependent manner and enhanced the intracellular concentration of DOX [146]. Quercetin, ferulic acid, and CAPE may also influence the MDR of cancer cells by inhibiting P-gp expression [142]. Components of red propolis (propolone B and propolonone A) displayed antiproliferative activities against glioma cells (U-251), breast cancer cells (MCF-7), and prostate cancer cells (PC-3). Propolone B and propolonone A also inhibited the proliferation of multidrug-resistant ovarian cancer cells line NCI-ADR/RES and overcame more efficiently than doxorubicin [147]. Frion-Herrera and colleagues discovered the chemosensitizing activity of Cuban propolis (CP) and its main compound (nemorosone) in doxorubicin-resistant colon cancer cells (LoVo). Combination of DOX and propolis extracts or Nem decreased viability of LoVo WT and DOX-resistant cells. All combined treatments increased reactive oxygen species production compared to control and single treatments in wild-type and resistant LoVo cells [145].

Propolis is usually well tolerated by cancer patients in clinical trials. Moreover, the patients appreciated the fact that they use a natural and well-known substance [140]. However, propolis may also be allergenic and may cause gastric problems [140,148]. A certain limitation in the use of propolis is also the highly variable chemical composition, which depends on the botanical origin and extraction methods. As a result, different propolis extracts are characterized by different biological activities [145]. Therefore, it is necessary to develop standarization methods, which will allow to combine the presence of specific compounds with biological activity and to develop recommendations for the use of different types of propolis [149].

## 5. Conclusions

Propolis is one of the most interesting substances produced by honey bees. Its antibacterial, antifungal, and immunomodulatory properties have been known since antiquity. Propolis also has an anticancer effect. Currently conducted research is aimed at understanding the molecular mechanisms by which propolis and its components inhibit carcinogenesis.

The compounds contained in propolis inhibit multiple signaling pathways crucial for cancer initiation, progression, and metastasis, such as PI3k/AKT/mTOR, NFκB, JAK-STAT, TLR4, VEGF, TGFβ, and intrinsic and extrinsic apoptosis pathways. Through the above-mentioned pathways, propolis can induce apoptosis, cell cycle arrest, and reduce proliferation, viability, invasion, migration, and chemoresistance of cancer cells. Therefore, we tried to gather molecular evidence on how propolis is able to inhibit many types of cancers, which may lead to the development of new anti-cancer drugs or supplements that can reduce the side effects of chemotherapy and radiotherapy.

## Figures and Tables

**Table 1:** Propolis compounds with anticancer activity (in vitro and in vivo models).

Compound Name, IUPAC Name; Concentration Used	Model	Property	Chemical Structure	Reference
Flavonoids, flavanones, flavones and flavonols				
Chrysin (5,7-dihydroxy-2-phenylchromen-4-one) 50 μM 5, 25, 50, 80 µg/mL	DU145 and PC-3 cells CAL-27 cells	induction of apoptosis	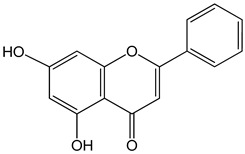	[25,26]
Galangin (3,5,7-trihydroxy-2-phenylchromen-4-one) 0–40 μM 0–40 μM 10, 20 and 30 mg/kg	mice bearing B16F1 TU212, M4e, HBE, HEP-2 RTE, and HHL-5 cells BALB/c nude mice	induction of apoptosis induction of apoptosis and inhibition of migration	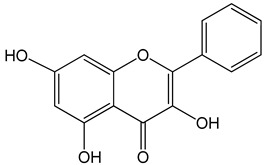	[27,28]
Genistein (5,7-dihydroxy-3-(4-hydroxyphenyl)chromen-4-one) 0–120 μM	LNCaP cells; mouse BALB/c 3T3 and SVT2 (SV40-transformed BALB/c 3T3) fibroblasts	inhibition of cell cycle	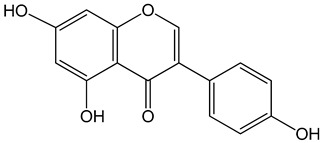	[3]
Nymphaeol A/Propolin C ((2S)-2-(3,4-dihydroxyphenyl)-6-[(2E)-3,7-dimethylocta-2,6-dienyl]-5,7-dihydroxy-2,3-dihydrochromen-4-one) 5–20 μM 2.5–20 μM	A549 cells A549 and HCC827 cells	anti-angiogenic activity, inhibition of proliferation inhibition of migration and invasion	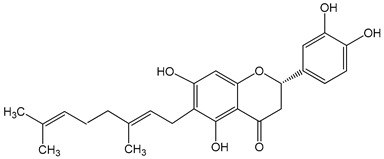	[29,30]
Nymphaeol C ((2S)-2-[2-[(2E)-3,7-dimethylocta-2,6-dienyl]-3,4-dihydroxyphenyl]-5,7-dihydroxy-6-(3-methylbut-2-enyl)-2,3-dihydrochromen-4-one) 5–20 μM		anti-angiogenic activity, inhibition of proliferation	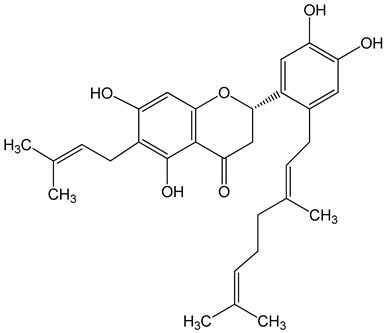	[29]
Vestitol (3-(2-hydroxy-4-methoxyphenyl)-3,4-dihydro-2H-chromen-7-ol) 0.37, 3.7, 37, and 370 μM	HeLa cells	cytotoxic effect	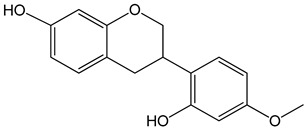	[31]
Aromatic acids and their derivatives				
Artepillin C ((E)-3-[4-hydroxy-3,5-bis(3-methylbut-2-enyl)phenyl]prop-2-enoic acid) 250 μM 100 μg/mL 0–150 μM	HT1080, A549, and U2OS cells BALB/c nude mice AGP-01 and HeLa cells CWR22Rv1 cells	inhibition of proliferation cytotoxic effect autophagy inhibition	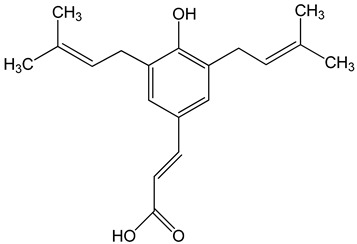	[32,33,34]
Baccharin ((1R,3S,4S,6R,9R,13S,15R,16S,19R,20E,22Z,26R,27S,28S)-16-hydroxy-19-[(1R)-1-hydroxyethyl]-6,15,27-trimethylspiro [2,5,11,14,18,25-hexaoxahexacyclo [2 4.2.1.03,9.04,6.09,27.013,15]nonacosa-20,22-diene-28,2′-oxirane]-12,24-dione) 0–150 μM	CWR22Rv1 cells	autophagy inhibition	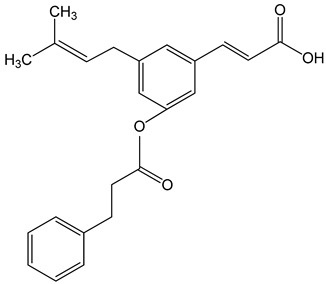	[34]
Caffeic acid ((E)-3-(3,4-dihydroxyphenyl)prop-2-enoic acid) 50 and 100 μM 65, 130, 190 µg/mL 30 μg/mL, 200 μg/mL 12.5 μM, 1 mM, 50 μM, 100 mg/kg, 20 mg/kg	MDA-MB-231 cells CAL-27 cells Hep3, SK-Hep1, HepG2 cells	cell cycle arrest in a dose- and time-dependent manner apoptosis activation inhibition of angiogenesis, apoptosis activation	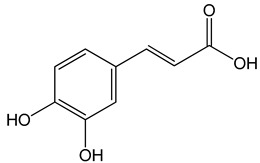	[26,35,36]
Caffeic acid phenylethyl ester (2-phenylethyl (E)-3-(3,4-dihydroxyphenyl)prop-2-enoate) 0.005–0.1 mg/mL 0.5–500 µM 10 mg/kg/day 15 mg/kg	AGS, HCT116, HT29, YD15, HSC-4, HN22, MCF-17, MDA-MB-231, MDA-MB-468, A549, HT1080, G361, U2OS, LNCaP, PC-3, DU145, Hep2, SAS, OECM-1, TW01, TW04, SW620, H460 and PANC-1 cells Balb/c nude mice BALB/c AnM-Foxn-1 mice	inhibition of proliferation, migration and invasion, pro-apoptotic activity anti-metastatic activity	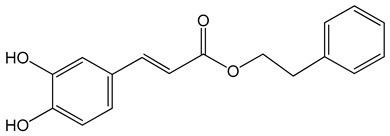	[3,35,37,38,39,40,41,42,43,44,45]
Ferulic acid ((E)-3-(4-hydroxy-3-methoxyphenyl)prop-2-enoic acid) 50, 100, 150 µg/mL	CAL-27 cells	apoptosis activation	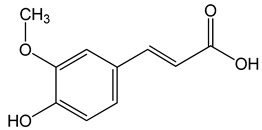	[26]
p-coumaric acid ((E)-3-(4-hydroxyphenyl)prop-2-enoic acid) 100 μg/mL 70, 140, 210 µg/mL	AGP-01 and HeLa cells CAL-27 cells	cytotoxic effect apoptosis activation	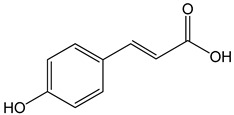	[26,33]
Other				
Frondoside A (sodium;[(3R,4R,5R,6S)-6-[(2S,4S,6S,12R,13R,18R)-4-acetyloxy-2,6,13,17,17-pentamethyl-6-(4-methylpentyl)-8-oxo-7-oxapentacyclo[10.8.0.02,9.05,9.013,18]icos-1(20)-en-16-yl]oxy]-5-[(2S,3R,4S,5S,6R)-5-[(2S,3R,4S,5R)-4-[(2S,3R,4S,5R,6R)-3,5-dihydroxy-6-(hydroxymethyl)-4-methoxyoxan-2-yl]oxy-3,5-dihydroxyoxan-2-yl]oxy-4-hydroxy-6-methyl-3-[(2S,3R,4S,5R)-3,4,5-trihydroxyoxan-2-yl]oxyoxan-2-yl]oxy-4-hydroxyoxan-3-yl] sulfate) 0.3–1.2 μM	A549 cells	anti-angiogenic activity, inhibition of proliferation	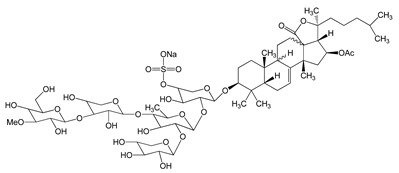	[29]
Nemorosone ((1R,5R,7S)-1-benzoyl-4-hydroxy-8,8-dimethyl-3,5,7-tris(3-methylbut-2-enyl)bicyclo[3.3.1]non-3-ene-2,9-dione) 5–50 μM	HT-29 and THP-1 cells	inhibition of migration and proliferation	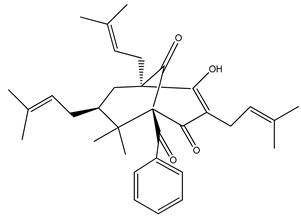	[46]

## Data Availability

Data sharing not applicable.
